# Supplementary material for: Biotin-Decorated PAMAM G4.5 Dendrimer Nanoparticles to Enhance the Delivery, Anti-Proliferative, and Apoptotic Effects of Chemotherapeutic Drug in Cancer Cells
Source: Pharmaceutics. 2020 May 11;12(5):443. doi: 10.3390/pharmaceutics12050443 (PMC7284937; doi:10.3390/pharmaceutics12050443)
Supplement: Supplementary file 1 [file pharmaceutics-12-00443-s001.pdf]

pharmaceutics

Article

Biotin-Decorated PAMAM G4.5 Dendrimer Nanoparticles to Enhance the Delivery, Anti-Proliferative, and Apoptotic Effects of Chemotherapeutic Drug in Cancer Cells

Endiries Yibru Hanurry [1], Tefera Worku Mekonnen [1], Abegaz Tizazu Andrgie [1], Haile Fentahun Darge [1], Yihenew Simegniew Birhan [1], Wei-Hsin Hsu [1], Hsiao-Ying Chou [1], Chih-Chia Cheng [1,2,*], Juin-Yih Lai [1,2,3] and Hsieh-Chih Tsai [1,2,3,*]

The standard curve of gemcitabine

The drug loading and encapsulation efficiency of gemcitabine was estimated by UV -Visible spectrophotometer using standard curve at 269 nm wavelength

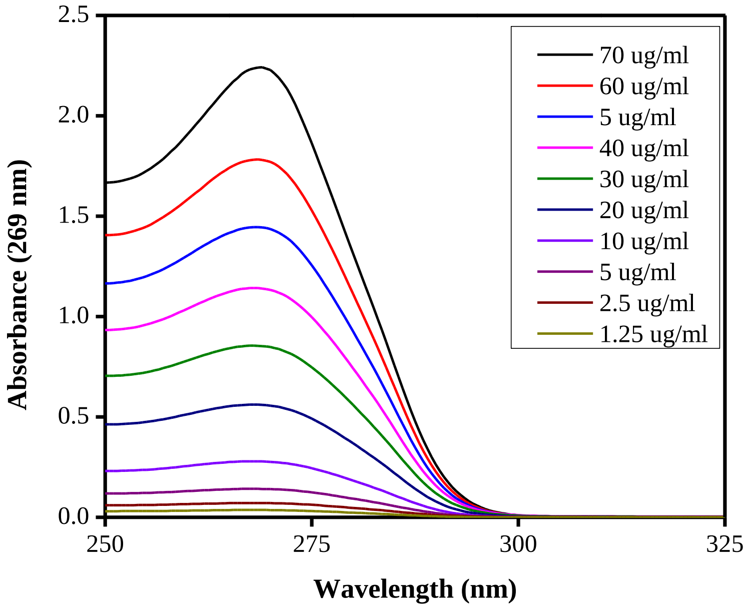

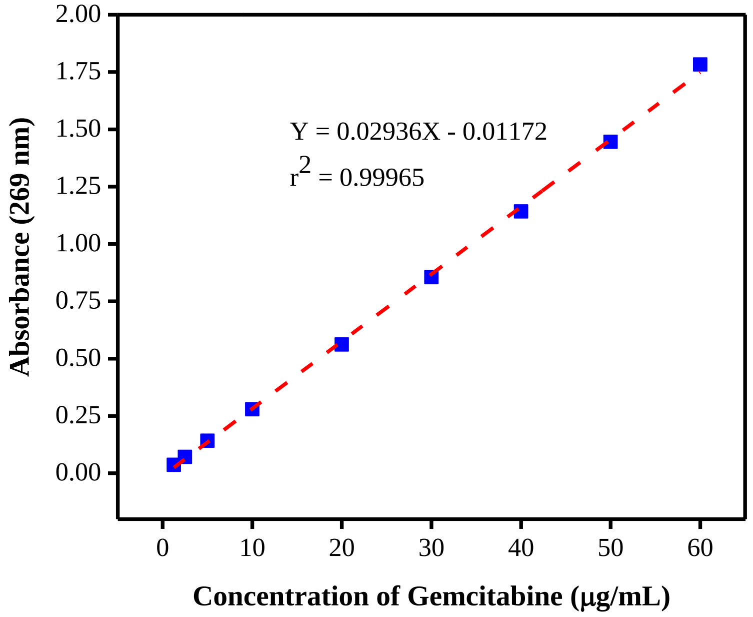

Figure S1. UV- Visible spectrophotometer study of gemcitabine. A) The absorbance of free gemcitabine at different concentration and B) The standard curve of free gemcitabine in PBS.

UV –Visible spectra of Fluorescein isothiocyanate (FITC)

PG4.5 –DETA-Biotin was tagged with FITC probe and the conjugation was characterized with UV-Visible spectrophotometer at 492 nm wavelength.

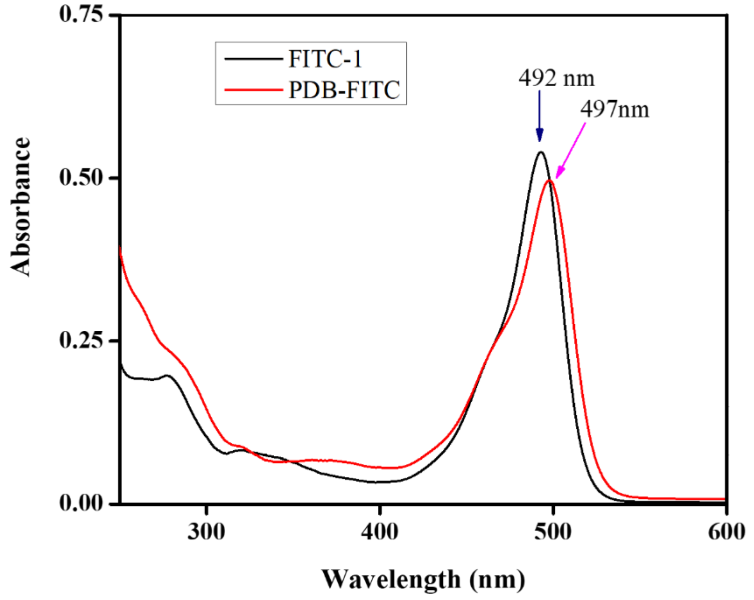

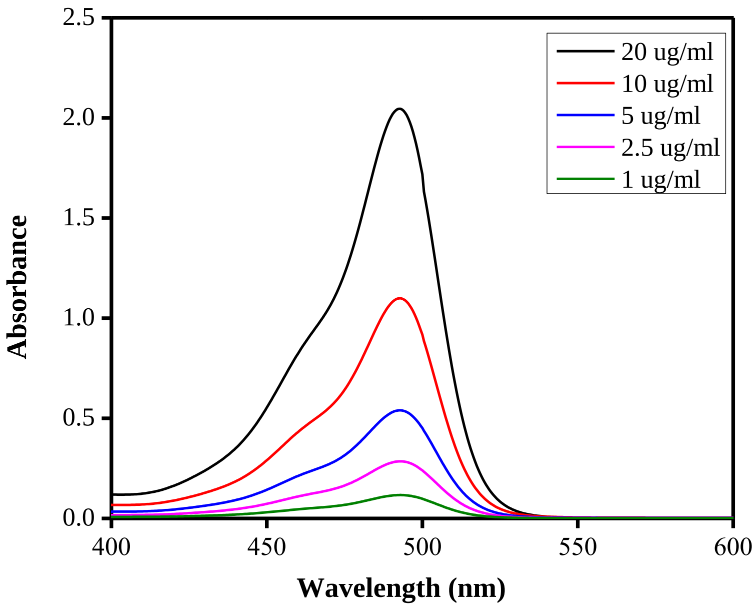

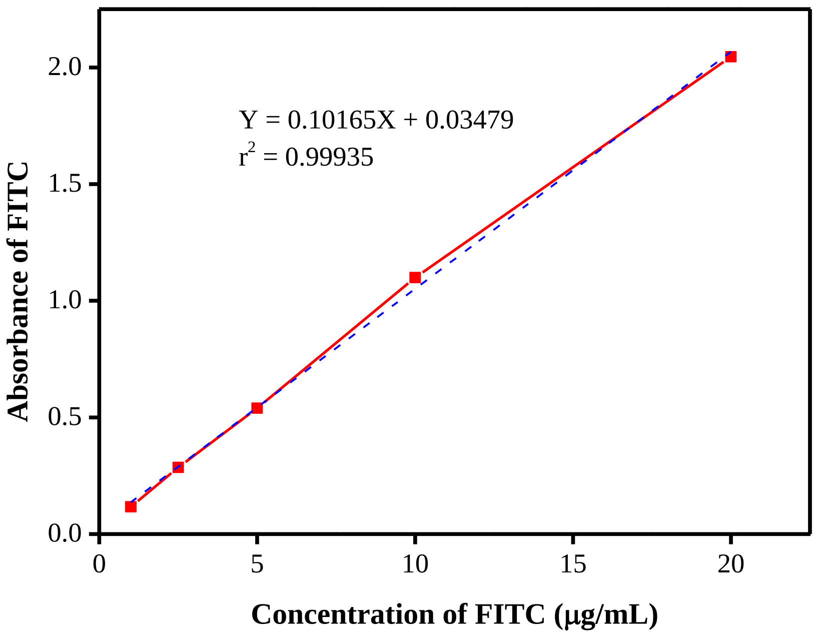

Figure S2. UV- Visible spectrophotometer study of FITC. A) The full adsorption wavelength spectrum of FITC and the PG4.5-DETA-Biotin B) The absorbance of FITC for different concentration and C) The standard curve of FITC in PBS.

© 2019 by the authors. Submitted for possible open access publication under the terms and conditions of the Creative Commons Attribution (CC BY) license (http://creativecommons.org/licenses/by/4.0/).
